# Immune and Iron Metabolism Responses During the Acute Phase of Experimental Hookworm Infection in the *Ancylostoma ceylanicum*-Hamster Model

**DOI:** 10.1007/s11686-026-01267-4

**Published:** 2026-05-04

**Authors:** William Pereira Alves, Vivian Jordania da Silva, Élida Mara Leite Rabelo, Luis Fernando Viana Furtado

**Affiliations:** 1 Universidade Federal de Minas Gerais, Hospital das Clínicas, Avenida Professor Alfredo Balena, 110, Santa Efigênia, Belo Horizonte, Minas Gerais CEP 30130-100 Brazil; 2 Prefeitura Municipal de Sabará, Centro de Controle de Zoonoses, Avenida Charles Gonort, Rosario I, Sabará, Minas Gerais CEP 34505620 Brazil; 3 Departamento de Parasitologia, Instituto de Ciências Biológicas, Universidade Federal de Minas Gerais, Avenida Presidente Antônio Carlos, 6627, Pampulha, Belo Horizonte, Minas Gerais CEP 31270-901 Brazil; 4 Departamento de Análises Clínicas e Toxicológicas, Faculdade de Farmácia, Universidade Federal de Minas Gerais, Avenida Presidente Antônio Carlos, 6627, Pampulha, Belo Horizonte, Minas Gerais CEP 31270-901 Brazil

**Keywords:** *Ancylostoma ceylanicum*, Hookworm infection, Iron metabolism, Hepcidin, Immune response, Experimental model

## Abstract

Hookworm infections continue to impose a substantial burden on human and animal health, but the early host responses that influence parasite establishment are not fully characterized. Experimental models that reproduce key features of hookworm biology and host-parasite interactions remain essential for advancing translational research. In this study, we examined hematological, biochemical, immunological, and parasitological parameters during the acute phase of experimental hookworm infection using the *Ancylostoma ceylanicum*-*Mesocricetus auratus* model, a small-animal system widely employed for mechanistic studies of hookworm infection. Animals were evaluated at 7 and 20 days post-infection. Hematological indices and serum iron concentrations did not differ between infected and control groups during the acute phase. In contrast, infected animals showed increased splenic mass at 20 days post-infection, indicating immunological activation. Hepatic hepcidin expression was markedly reduced, suggesting an early alteration in systemic iron regulation. Analysis of inflammatory mediators revealed selective modulation of cytokine expression, with reduced interleukin-6 transcript levels at 20 days post-infection, whereas tumor necrosis factor alpha expression remained unchanged. Parasitological analyses demonstrated progressive parasite establishment, with fecal egg output detected from 14 days post-infection and reaching approximately 300 eggs per gram by day 18, consistent with the onset of patency. Taken together, these data indicate that acute hookworm infection induces coordinated changes in immune responses and iron metabolism before the development of overt hematological alterations.

## Introduction

Hookworm infection remains a major public health concern, particularly in low- and middle-income countries, where it is among the most prevalent neglected tropical diseases [5]. In humans, infection is mainly caused by three species, listed here according to their global prevalence: *Necator americanus*, *Ancylostoma duodenale*, and *Ancylostoma ceylanicum* [19, 30]. Several animal species also act as natural hosts for hookworms, especially dogs and cats, which are commonly infected by *A. caninum*, *A. braziliense*, and *A. tubaeforme* [21]. These hematophagous nematodes attach to the intestinal mucosa and feed on host blood, resulting in chronic intestinal blood loss [17]. As a consequence, hookworm disease remains one of the leading causes of iron-deficiency anemia worldwide [4, 27].

The development of hookworm-associated anemia reflects the interaction between iron metabolism, erythropoiesis, and host immune responses [3, 10]. Blood loss induces compensatory mechanisms aimed at maintaining iron availability and red blood cell production, including the regulation of hepcidin (HAMP), erythroferrone (ERFE), and erythropoietin (EPO) [18, 22]. In parallel, inflammatory cytokines may affect both erythropoiesis and iron distribution, contributing to anemia of inflammation [34]. How these pathways are coordinated during hookworm infection, particularly at early stages, remains incompletely understood.

Previous studies have largely focused on the chronic phase of *A. ceylanicum* infection and have demonstrated sustained alterations in iron homeostasis and immune regulation [12]. In contrast, the early events that precede these long-term changes are less well characterized. The acute phase represents a critical window during which the host initially responds to infection, activating immune and metabolic pathways that may influence the subsequent development of anemia.

In the present study, we investigated the regulation of iron metabolism and inflammatory mediators during the acute phase of experimental hookworm infection. These findings complement previous observations from the chronic phase and provide a more integrated view of the mechanisms linking parasite-induced blood loss to anemia. To address this question, we used the *A. ceylanicum*-*Mesocricetus auratus* experimental model, which supports intestinal establishment and patent infection and has been widely applied in mechanistic studies of hookworm disease [12, 29]. Although parasite establishment in hamsters is influenced by host age, this system remains suitable for controlled investigation of early host immunometabolic responses.

## Material and Methods

### Ethics Statement and Experimental Design

All experimental procedures were conducted in accordance with the ethical principles established by the Brazilian National Council for the Control of Animal Experimentation (CONCEA) and national legislation governing the scientific use of animals (Law N^o^. 11.794/2008). The study protocol was approved by the local Institutional Animal Care and Use Committee (Protocol No. 13/2017).

Female golden hamsters (*M. auratus*), four to six weeks of age, were individually identified and randomly allocated to experimental groups according to infection status and sampling time. Animals were orally inoculated by gavage with 100 third-stage larvae (L3) of *A. ceylanicum*.

Two experimental time points were selected to evaluate the acute phase of infection: 7 days post-infection (dpi), corresponding to early parasite establishment, and 20 dpi, representing the transition to egg production. At each time point, infected and non-infected groups were analyzed as follows: NI7 (non-infected, 7 dpi; n = 7), AI7 (infected, 7 dpi; n = 7), NI20 (non-infected, 20 dpi; n = 7), and AI20 (infected, 20 dpi; n = 7).

Fecal samples were collected starting at 10 dpi and subsequently every two days to monitor parasite establishment and egg shedding. Egg counts were performed using a McMaster chamber, with three independent readings per group, following the method described by Gordon and Whitlock [14].

Animals were euthanized under deep anesthesia by intraperitoneal administration of xylazine hydrochloride (45 mg/kg) and ketamine (240 mg/kg) (Sigma-Aldrich, USA). Blood and tissue samples were collected immediately after euthanasia for hematological, biochemical, immunological, and molecular analyses.

After euthanasia, the abdominal cavity was opened and the small intestine was excised. Based on previous studies indicating the absence of adult worms at 7 dpi [7, 13, 29], intestines from NI7 and AI7 animals were opened longitudinally and processed using a modified Baermann technique [33]. Recovered larvae were collected after two hours of sedimentation and quantified. For NI20 and AI20 groups, intestines were similarly opened, and adult worms were recovered manually using fine forceps and counted under a stereomicroscope.

### Hematological, Biochemical, and Morphological Assessments

Blood samples were obtained from the sublingual venous plexus. An aliquot of 100 μL of whole blood containing EDTA was used for automated hematological analysis, including hemoglobin concentration, hematocrit, and red blood cell count, using a volumetric impedance hematology analyzer (Abacus Júnior Vet, Austria).

For serum analysis, approximately 200 μL of blood was collected, allowed to clot at room temperature for 20 min, and centrifuged at 10,000 × g for 10 min. Serum iron concentrations were determined using a commercial colorimetric kit (Bioclin, Brazil) according to the manufacturer’s instructions. Absorbance was measured using an Epoch™ microplate spectrophotometer (BioTek, United States).

After euthanasia, spleens were excised, cleaned of surrounding tissue, and weighed on an analytical balance. Spleen mass was used as an indirect indicator of hematopoietic and immunological activity.

### Tissue Collection and Analysis of Gene Expression

The entire mesenteric lymph node complex and approximately 200 mg of the left hepatic lobe were collected from each animal. Tissues were immediately immersed in RNAlater™ Stabilization Solution (Invitrogen, United States) and stored at 4 °C until processing. Total RNA was extracted using an Omni TH homogenizer and TRIzol™ reagent (Invitrogen, United States) according to the manufacturer’s instructions, followed by DNase treatment using the TURBO DNA-free™ kit (Ambion, United States).

Complementary DNA (cDNA) was synthesized from 1 μg of total RNA in a final volume of 20 μL using the High-Capacity cDNA Reverse Transcription Kit (Applied Biosystems, United States). Negative control reactions without reverse transcriptase were included to verify the absence of genomic DNA contamination.

Gene expression was quantified by quantitative polymerase chain reaction (qPCR) using a StepOnePlus™ Real-Time PCR System (Applied Biosystems, United States). Reactions were performed in triplicate in a final volume of 10 μL containing PowerUp™ SYBR® Green PCR Master Mix (Applied Biosystems, United States), 0.3 μM of each primer, and 2 μL of cDNA. Amplification was carried out using a two-step protocol consisting of an initial denaturation at 95 °C for 10 min, followed by 40 cycles of denaturation at 95 °C for 15 s and combined annealing/extension at 60 °C for 60 s. A melt curve analysis was performed at the end of each run to confirm amplification specificity.

Primer sequences are provided in Table 1 and were previously described by Furtado et al. [12]. Relative gene expression was calculated using the 2^⁻ΔΔCt^ method as described by Livak and Schmittgen [24], after normalization to hypoxanthine–guanine phosphoribosyltransferase (HPRT). Interleukin-6 (IL-6) and tumor necrosis factor alpha (TNF-α) expression were assessed in mesenteric lymph nodes, whereas HAMP expression was evaluated in liver samples.

**Table 1:** Primer sequences for genes analyzed by qPCR

Gene’s acronym	Primer sequence (5’-3’)
HAMP	F: CCT GTT TCT TGA TCC TCC TC R: CTG TAG TGC TTC AGG CTG TC
TNF-α	F: TGA GCC ATC GTG CCA ATG R: AGC CCG TCT GCT GGT ATC AC
IL-6	F: CAC CAT CAA AAC CCT AAG TCA GAT C R: TGG GCT AGG CGT GAC TAT TTT ATC

### Statistical Analysis

Results are expressed as mean ± standard deviation. Data distribution was assessed using the Shapiro–Wilk test, and homogeneity of variances was evaluated using Levene’s test prior to the application of parametric analyses. When assumptions of normality and equal variances were met, data were analyzed using two-way analysis of variance (ANOVA) to evaluate the effects of infection status and time, followed by Tukey’s post hoc test when appropriate. In cases where parametric assumptions were not satisfied, the Mann–Whitney U test was applied for pairwise comparisons. Differences were considered statistically significant at *p* < 0.05. Statistical analyses were conducted using GraphPad Prism version 5.0 (GraphPad Software, United States).

## Results

### Hematological Parameters

**Fig. 1:**
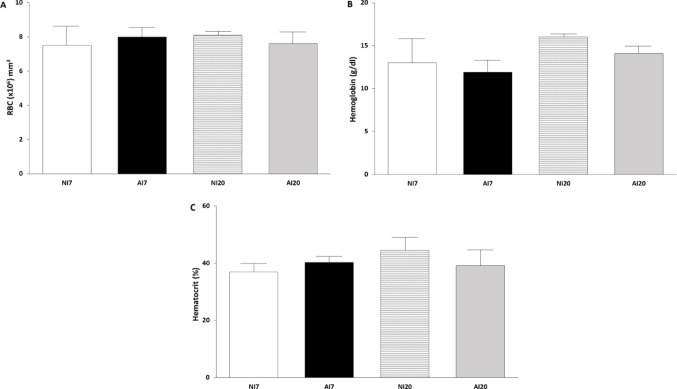
Hematological parameters in *Mesocricetus* auratus experimentally infected with *Ancylostoma ceylanicum* Hemoglobin concentration **A**, red blood cell count **B**, and hematocrit **C** in non-infected (NI) and infected (AI) hamsters at 7 and 20 days post-infection Data are shown as mean ± standard deviation (n = 7 per group) Statistical comparisons were performed only between infected and non-infected groups within each time point (NI7 vs. AI7 and NI20 vs. AI20) No comparisons were performed between time points

Hematological parameters remained unchanged during the acute phase of *A. ceylanicum* infection at both 7 and 20 dpi. No significant differences were observed in red blood cell count (Fig. 1A), hemoglobin concentration (Fig. 1B), or hematocrit (Fig. 1C) between infected and non-infected animals at either time point.

### Serum Iron Concentration and Spleen Weight

**Fig. 2:**
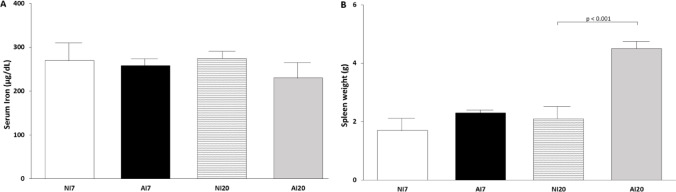
Serum iron concentration and spleen weight in *Mesocricetus auratus* experimentally infected with *Ancylostoma ceylanicum*. Serum iron concentration **A** and spleen weight **B** in non-infected (NI) and infected (AI) hamsters at 7 and 20 days post-infection Data are shown as mean ± standard deviation (n = 7 per group). Statistical comparisons were performed only between infected and non-infected groups within each time point (NI7 vs AI7 and NI20 vs AI20). *p* < 0.001 compared with the corresponding NI group

Serum iron concentrations did not differ between infected and non-infected groups at either 7 or 20 dpi (Fig. 2A). In contrast, spleen weight was significantly increased in infected animals at 20 dpi compared with the corresponding non-infected group (*p* < 0.001) (Fig. 2B). No differences in spleen weight were observed at 7 dpi.

### Hepatic Hepcidin (HAMP) Expression

**Fig. 3:**
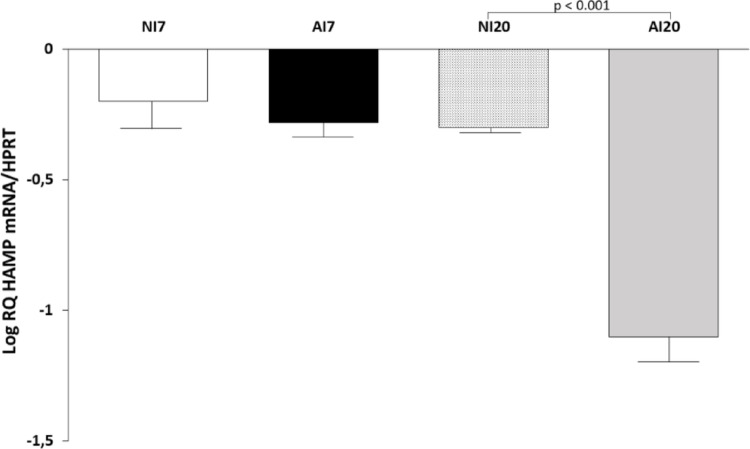
Hepatic expression of hepcidin (HAMP) mRNA in *Mesocricetus auratus* experimentally infected with *Ancylostoma ceylanicum*. Relative HAMP transcript levels expressed as log relative quantification normalized to hypoxanthine–guanine phosphoribosyltransferase (HPRT) in the liver of non-infected (NI) and infected (AI) hamsters at 7 and 20 days post-infection. Data are shown as mean ± standard deviation (n = 7 per group). Statistical comparisons were performed only between infected and non-infected groups within each time point (NI7 vs. AI7 and NI20 vs. AI20). *p* < 0.001 compared with the corresponding NI group

Hepatic expression of HAMP mRNA, expressed as relative quantification normalized to HPRT, did not differ between groups at 7 dpi (Fig. 3). At 20 dpi, HAMP expression was significantly reduced in infected animals compared with non-infected controls (*p* < 0.001).

### Expression of Inflammatory Cytokines

**Fig. 4:**
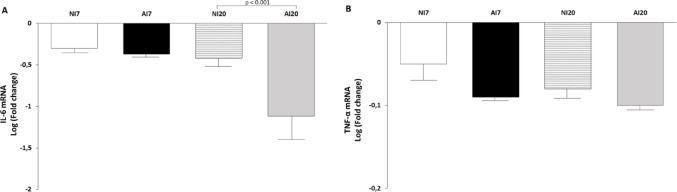
Expression of inflammatory cytokines in mesenteric lymph nodes of *Mesocricetus auratus* experimentally infected with *Ancylostoma ceylanicum*. Interleukin-6 **A** and tumor necrosis factor alpha **B** transcript levels normalized to hypoxanthine–guanine phosphoribosyltransferase (HPRT) in non-infected (NI) and infected (AI) hamsters at 7 and 20 days post-infection. Data are shown as mean ± standard deviation (n = 7 per group). Statistical comparisons were performed only between infected and non-infected groups within each time point (NI7 vs AI7 and NI20 vs AI20). *p* < 0.001 compared with the corresponding NI group

IL-6 mRNA expression did not differ between infected and non-infected animals at 7 dpi. At 20 dpi, IL-6 expression was significantly lower in infected animals than in non-infected controls (*p* < 0.001) (Fig. 4A). TNF-α expression showed no significant differences between groups at either time point (Fig. 4B).

### Parasite Burden and Fecal Egg Counts

Fecal egg counts were initiated at 10 dpi and performed every two days thereafter. No egg shedding was detected in animals euthanized at 7 dpi (NI7 and AI7). Egg excretion was first detected at 14 dpi in infected animals from the AI20 group, with a mean count of approximately 120 eggs per gram of feces. Egg output increased to an average of approximately 300 eggs per gram between 16 and 18 dpi.

Parasitological examination revealed a mean recovery of 62 larval forms per animal in AI7 hamsters. In AI20 animals, a mean of 74 adult worms per animal was recovered. No parasitic stages were detected in non-infected control groups (NI7 and NI20).

## Discussion

Hookworm infection remains a major public health concern, particularly in regions with inadequate sanitation and limited socioeconomic resources [23]. Iron-deficiency anemia is its most prominent clinical manifestation and is associated with impaired physical and cognitive development, reduced work capacity, and increased morbidity [10]. Understanding the mechanisms underlying parasite-induced anemia is therefore central to improving the clinical management and control of human hookworm disease.

The pathophysiological basis of hookworm-associated anemia has been extensively investigated in human populations and in experimental models [4, 7, 29]. Among these, the *A. ceylanicum-M. auratus* model has been widely employed because it supports intestinal establishment and patent infection under controlled conditions [12, 16, 26, 29, 32]. Although parasite development in hamsters is influenced by host age, with more consistent establishment in young animals, this system remains a well-characterized and reproducible platform for mechanistic studies of hookworm infection [9, 15].

Studies focusing on the chronic phase of experimental *A. ceylanicum* infection have demonstrated the development of sustained anemia, splenic erythropoietic expansion, and long-term modulation of iron metabolism [12]. However, the early events that precede these chronic alterations, particularly those occurring before the onset of patent infection, remain incompletely characterized.

In the present study, hematological parameters remained unchanged during the acute phase of *A. ceylanicum* infection. This observation is consistent with clinical and experimental evidence indicating that overt anemia typically develops during the chronic phase, when adult worms are established and sustained blood feeding occurs [6, 8, 28]. The preservation of hemoglobin concentration, hematocrit, and erythrocyte counts at 7 and 20 dpi indicates that compensatory mechanisms are sufficient to maintain red blood cell homeostasis during early infection.

Despite stable systemic iron concentrations, infected animals exhibited marked splenic enlargement at 20 dpi. In the context of hookworm infection, splenic enlargement has been associated with early hematopoietic adjustments and immune cell activation, processes that are also described in human helminth infections [2, 31, 35]. These findings suggest that compensatory responses to parasite-induced blood loss may be initiated before anemia becomes clinically detectable.

A key finding of this study is the significant reduction in hepatic HAMP expression at 20 dpi, occurring in the absence of measurable iron depletion. In humans, HAMP plays a central role in regulating iron availability during infection and inflammation [11, 20]. The concomitant reduction in IL-6 expression indicates that early HAMP suppression during hookworm infection may occur independently of classical inflammatory signaling. Such early modulation of iron-restrictive pathways may facilitate iron mobilization in anticipation of increased erythropoietic demand during later stages of infection [1].

The absence of changes in TNF-α expression further supports the notion that the acute phase of infection is characterized by limited systemic inflammation. This immunological profile is compatible with early host–parasite accommodation and has been described in human helminth infections prior to the establishment of chronic pathology [25]. Parasitological analyses showed that larval stages predominated at 7 dpi, whereas adult worms and egg shedding were detected by 20 dpi, coinciding temporally with the observed immunometabolic adjustments.

## Conclusions

Integration of acute and chronic infection data supports a temporal model of host adaptation to hookworm-induced blood loss. The acute phase is characterized by preserved hematological parameters, early splenic responses, and initial suppression of hepatic HAMP expression, whereas the chronic phase is marked by overt anemia, expanded erythropoiesis, and sustained modulation of iron metabolism [12]. These findings contribute to a clearer understanding of the early pathophysiological mechanisms underlying human hookworm-associated anemia and highlight the value of experimental infection models for elucidating host–parasite interactions relevant to disease progression and control.

## Data Availability

No datasets were generated or analysed during the current study.
